# Comparing Observed with Predicted Weekly Influenza-Like Illness Rates during the Winter Holiday Break, United States, 2004-2013

**DOI:** 10.1371/journal.pone.0143791

**Published:** 2015-12-09

**Authors:** Hongjiang Gao, Karen K. Wong, Yenlik Zheteyeva, Jianrong Shi, Amra Uzicanin, Jeanette J. Rainey

**Affiliations:** 1 Centers for Disease Control and Prevention, National Center for Disease Control and Prevention, Division of Global Migration and Quarantine, Atlanta, Georgia, United States of America; 2 Chenega Time Solutions, Chesapeake, Virginia, United States of America; Public Health Agency of Canada, CANADA

## Abstract

In the United States, influenza season typically begins in October or November, peaks in February, and tapers off in April. During the winter holiday break, from the end of December to the beginning of January, changes in social mixing patterns, healthcare-seeking behaviors, and surveillance reporting could affect influenza-like illness (ILI) rates. We compared predicted with observed weekly ILI to examine trends around the winter break period. We examined weekly rates of ILI by region in the United States from influenza season 2003–2004 to 2012–2013. We compared observed and predicted ILI rates from week 44 to week 8 of each influenza season using the auto-regressive integrated moving average (ARIMA) method. Of 1,530 region, week, and year combinations, 64 observed ILI rates were significantly higher than predicted by the model. Of these, 21 occurred during the typical winter holiday break period (weeks 51–52); 12 occurred during influenza season 2012–2013. There were 46 observed ILI rates that were significantly lower than predicted. Of these, 16 occurred after the typical holiday break during week 1, eight of which occurred during season 2012–2013. Of 90 (10 HHS regions x 9 seasons) predictions during the peak week, 78 predicted ILI rates were lower than observed. Out of 73 predictions for the post-peak week, 62 ILI rates were higher than observed. There were 53 out of 73 models that had lower peak and higher post-peak predicted ILI rates than were actually observed. While most regions had ILI rates higher than predicted during winter holiday break and lower than predicted after the break during the 2012–2013 season, overall there was not a consistent relationship between observed and predicted ILI around the winter holiday break during the other influenza seasons.

## Introduction

In the United States, influenza season typically begins in October or November, peaks in February, and tapers off in April, although the timing and duration vary from year to year [1]. The U.S. Centers for Disease Control and Prevention (CDC) assesses influenza activity using the National Influenza Sentinel Surveillance System for influenza-like-illness (ILINet) [2]. More than 2,900 ILINet sentinel providers in all 50 states, Puerto Rico, the District of Columbia, and the U.S. Virgin Islands report weekly visits for influenza-like illnesses (ILI), defined as fever (≥100°F [≥37.8°C]), plus cough and/or sore throat, in the absence of another known cause of illness.

There are a number of studies[3–5] suggesting that school closures, which temporarily change the social contact patterns of children, can reduce total illnesses and peak incidence of pandemic and seasonal influenza among school children. Among the general population, commonly observed holidays, such as the winter holiday break in late December to early January, also disrupt normal social mixing patterns. These periods provide unique opportunities to explore the relationship between ILI and temporary, atypical social patterns.

Assessing the impact of winter break on ILI is challenging. It is difficult to identify appropriate control series, and populations may not be similarly susceptible to influenza across regions. However, models that can be trained on longitudinal data to predict weekly ILI rates may be able to identify deviations from expected ILI patterns around a time period of interest.

The ARIMA method was first popularized by Box-Jenkins[6] for analyzing time-series data. Since then, they have been widely applied in fields such as engineering, economics, agriculture, meteorology, and infectious diseases, including influenza [7–9]. Unlike most generalized linear regression, in which model predictions are restricted to the range of predictors, ARIMA models forecast beyond the scope of model predictors using the recursive relationship between observations and error terms.

We apply the ARIMA method to compare observed and predicted weekly ILI around the winter holiday break period to explore patterns that may be associated with the holiday period.

## Methods

### Data Source

We obtained ILINet data for the 2003–2004 through 2012–2013 influenza seasons for each of 10 U.S. Health and Human Services (HHS) regions listed on the CDC website [2]. A U.S. HHS region is the geographic aggregation of 4–10 adjacent states or island areas. Each week, sentinel clinic providers in these regions report the number of clinic visits due to ILI, as well as the overall number of clinic visits [10]. The ILI rate (number of clinic visits due to ILI/total number of visits across the state’s sentinel clinics) is weighted according to the state population and aggregated to the regional level.

### Data Analysis

#### Winter Holiday Break

Because the beginning and ending dates of the winter holiday break may vary by geography and year, we approximated this using website announcements made by 4,297 public schools in Tennessee and North Carolina during the 2012–2013 influenza season (data originally collected for a different project). We found that most school districts started the winter holiday break during the week of Christmas, or the Thursday and Friday of the previous week, and ended the break on the first Monday after the New Year. Therefore, we approximated the disruption to normal social mixing patterns and healthcare-seeking behavior during the holiday break as the 2-week period that covered New Year’s Day.

#### Defining ILINet Surveillance Weeks

The ILI weeks in this research were defined in the same way as they were for the CDC’s *Morbidity and Mortality Weekly Report* (MMWR) [11]. The first day of any week is Sunday. Week # 1 is the first week of the year having at least four days in the calendar year. Using this definition, years 2003 and 2008 consisted of 53 weeks in our analysis.

In addition to the first week and last week that encompass the New Year, we also compared observed and predicted ILI rates for weeks 44 to week 8 for each influenza season from 2004–2005 to 2012–2013 for the purpose of determining whether we can observe similar interruptions of ILI trend in these influenza weeks. Therefore, our analysis included a total of 1,530 unique (17 weeks x 9 seasons x 10 regions) predictions. However, for years 2003 and 2008, the starting week for our data analysis was week 45 rather than week 44, since years 2003 and 2008 had 53 weeks. In addition, each 17-week period was aligned with the 17-week periods from all other seasons.

#### Auto-Regressive Integrated Moving Average Method

To forecast ILI rates for each week-year-region combination, we used the ARIMA (*p*, *d*, *q*) method, in which *p* represents the number of autoregressive terms (i.e., number of previous observations on which the current observation linearly depends), *d* is the order of differencing, and *q* is the number of lagged forecast errors in the prediction equation (i.e., number of preceding estimation errors are taken into account when estimating the next time-series value). For example, to forecast the ILI rate for week 52 in 2004, we used all the data points in 2003, plus weeks 1–51 in 2004, as a training set to determine the proper parameters in the ARIMA model using model identification, model diagnosis, and forecasting [12]. In the identification step, we selected parameters *p*, *d*, *q* using Bayesian information criterion (BIC), which assigns a penalty according to the number of parameters in the model. In the model-diagnosis step, we used the Ljung-Box statistical test and quantile-quantile plot to check the time-series assumptions. We also applied the test outlined by Osborn et al [13] to check if seasonality terms should be included in the ARIMA model. Finally, in the forecasting step, we applied the model identified in the above steps to predict the weekly ILI rate and computed 95% prediction intervals by bootstrapping the model error 5,000 times.

We repeated these steps, as described above, for each combination of the 17 weeks (weeks 44 to week 8), nine influenza seasons, and 10 HHS regions. To generate the forecasting series for a specific week in one influenza season, we replaced the observed ILI rate for this week in all previous years with the predicted ILI rate to avoid the potential carryover effect by the actual ILI data in the prediction. For example, to forecast the ILI rate for week Y in influenza season 2005, we replaced the observed week Y ILI rates in 2003 and 2004 with ARIMA model-predicted values. Because there were not enough data points to have reliable predictions for the last week of 2003 and first week of 2004, these weeks are excluded from the results presented.

All analyses were performed using package Forecast 4.06 [14] in R 3.0.1 [15] and PROC ARIMA in SAS 9.3 for Windows 7 (SAS Institute, Cary NC). Because the ILI surveillance data are publicly available and include summary data only, this research was not subject to CDC institutional review board (IRB) review.

## Results

We present the last and first week predictions in the main article in Tables 1 and 2, Tables 3 and 4. All other results from weeks 44–51 and weeks 2–8, plus model-fitting procedures, are reported in the supplemental material. We summarize our results into three categories in this section: 1) predicted ILI rates lower than observed; 2) predicted ILI rates higher than observed; and 3) model predictions at peak and after peak.

**Table 1:** Last Week ILI rate prediction for HHS regions 1–5 from influenza seasons 2004–2005 to 2012–2013.

Influenza Season	HHS1			HHS2			HHS3			HHS4			HHS5		
2004–2005	(1,0,2)	2.27(1.66,2.69)	2.27	(1,0,0)	3.90(2.79,5.56)	3.82	(1,0,0)	3.26(2.36,4.56)	4.00	(2,0,2)	1.78(1.20,2.65)	1.73	(1,0,2)	1.69(1.10,2.71)	2.11
2005–2006	(1,0,2)	0.86(0.27,1.47)	1.35	(1,0,0)	2.51(1.47,3.65)	2.65	(1,0,0)	2.42(1.52,3.97)	3.63	(2,0,2)	1.50(0.99,2.29)	2.01	(2,0,2)	2.21(1.65,3.13)	2.39
2006–2007	**(1,0,2)**	**0.89(0.34,1.41)**	**1.59**	(1,0,0)	2.74(1.71,3.88)	2.67	(1,0,0)	2.97(2.06,4.48)	3.70	**(2,0,2)**	**3.82(3.36,4.56)**	**3.01**	(2,0,2)	2.40(1.87,3.35)	2.67
2007–2008	(1,0,2)	0.93(0.40,1.47)	1.19	(1,0,0)	1.75(0.76,2.80)	1.96	(1,0,0)	2.07(1.18,3.40)	2.62	(1,0,2)	1.94(1.43,2.61)	2.06	(2,0,2)	1.86(1.34,2.46)	1.98
2008–2009	(1,0,2)	1.03(0.57,1.62)	1.05	(2,0,1)	1.69(0.89,2.81)	2.03	(1,0,0)	2.45(1.62,3.76)	2.37	(2,0,2)	2.01(1.54,2.76)	1.58	(2,0,2)	1.45(0.94,2.21)	1.26
2009–2010	(1,0,3)	1.00(0.46,1.84)	1.13	(2,1,1)	2.34(1.43,3.59)	2.32	(1,0,3)	2.73(1.80,4.24)	2.34	(3,1,0)	2.70(1.89,3.61)	2.32	(2,0,2)	2.01(1.46,2.78)	1.91
2010–2011	(1,0,2)	1.07(0.53,1.90)	1.13	(1,0,0)	3.99(3.10,5.30)	4.48	(1,0,0)	2.17(1.29,3.66)	2.61	**(2,0,2)**	**4.77(4.20,5.73)**	**4.00**	(2,0,2)	1.59(1.07,2.36)	1.70
2011–2012	(2,0,2)	0.93(0.43,1.64)	1.01	(2,0,2)	1.23(0.35,2.45)	1.18	(2,0,1)	1.87(1.00,3.01)	2.14	(1,0,2)	2.07(1.46,3.07)	2.29	(2,0,2)	1.34(0.84,2.11)	1.54
2012–2013	**(2,0,2)**	**2.81(2.31,3.46)**	**3.93**	**(2,0,2)**	**3.82(2.9,5.03)**	**5.51**	**(2,0,2)**	**4.55(3.7,5.89)**	**7.13**	**(1,0,2)**	**4.71(4.11,5.54)**	**6.33**	**(2,0,2)**	**4.65(4.11,5.25)**	**5.71**

**Table 2:** Last Week ILI rate prediction for HHS regions 6–10 from influenza seasons 2004–2005 to 2012–2013.

Influenza Season	HHS6			HHS7			HHS8			HHS9			HHS10		
2004–2005	(2,0,2)	2.64(1.32,3.94)	2.36	(1,0,2)	1.91(0.85,3.49)	1.66	(1,0,0)	1.13(0.03,2.94)	1.60	**(2,0,2)**	**3.12(1.92,4.55)**	**4.69**	(1,0,0)	1.55(0.22,3.59)	1.86
2005–2006	(2,0,2)	5.15(3.77,6.52)	5.99	(1,1,3)	1.05(-0.06,2.10)	1.02	(1,0,2)	2.90(2.16,3.83)	2.80	**(2,0,2)**	**7.67(6.41,9.11)**	**5.54**	(1,0,0)	2.31(1.27,4.33)	2.50
2006–2007	(2,0,2)	4.79(3.50,6.25)	5.67	(1,1,3)	2.36(1.37,3.30)	2.75	(1,0,0)	0.74(-0.09,2.41)	1.45	(1,0,0)	2.54(1.37,4.17)	2.48	(1,0,0)	1.86(0.82,3.33)	2.23
2007–2008	(2,0,2)	3.05(1.86,4.56)	3.97	(1,1,3)	1.07(0.21,1.89)	1.74	(1,0,0)	1.12(0.35,2.18)	1.48	**(1,0,0)**	**2.36(1.20,3.58)**	**3.65**	(1,0,0)	2.06(0.88,4.03)	2.76
2008–2009	(2,0,2)	3.12(1.99,4.71)	4.33	(1,0,2)	0.54(-0.38,1.53)	0.35	(1,0,2)	0.61(-0.12,1.53)	0.38	(1,0,0)	1.95(0.74,3.34)	3.15	(1,0,0)	1.68(0.43,3.69)	2.11
2009–2010	(3,0,0)	3.85(2.67,5.63)	3.66	(1,0,2)	1.94(1.11,3.11)	1.94	(1,0,2)	1.13(0.34,2.39)	1.24	(1,0,0)	3.92(2.68,5.29)	4.40	(0,1,0)	1.57(-0.04,3.5)	1.99
2010–2011	(2,0,2)	4.12(3.00,5.75)	3.78	(1,1,3)	2.17(1.17,3.06)	2.29	(1,0,2)	0.92(0.18,1.77)	0.99	(2,0,2)	4.05(2.96,5.31)	4.17	(1,0,0)	1.30(0.19,3.14)	2.35
2011–2012	(2,0,2)	2.33(1.15,3.91)	2.29	(1,0,2)	1.56(0.68,2.60)	1.89	(1,0,2)	1.06(0.45,1.91)	1.11	(1,0,0)	2.97(1.75,4.17)	3.69	(1,0,0)	1.20(0.14,2.91)	1.25
2012–2013	**(2,0,2)**	**7.42(6.23,8.92)**	**9.26**	**(1,0,2)**	**4.24(3.41,5.47)**	**6.72**	(1,0,3)	3.33(2.70,4.14)	4.10	**(1,0,0)**	**2.96(1.94,4.25)**	**4.83**	**(1,0,0)**	**1.53(0.54,3.12)**	**3.39**

Note: 1) Auto-regressive integrated moving average (ARIMA [*p*, *d*, *q*]) method, in which *p* represents the number of auto-regressive terms, *d* is the number of non-seasonal differences, and *q* is the number of lagged forecast errors in the prediction equation; 2) 95% prediction intervals were calculated by bootstrapping the model error 5,000 times; 3) the point estimate and prediction interval were bolded if the observed ILIs rate were not covered by the 95% prediction interval; and 4) last week is defined as week 53 for influenza season 2008–2009 and week 52 for other influenza seasons.

**Table 3:** Week 1 ILI rate prediction for HHS regions 1–5 from influenza seasons 2004–2005 to 2012–2013.

	HHS1			HHS2			HHS3			HHS4			HHS5		
2004–2005	(1,0,2)	2.42(1.82,2.84)	2.12	(1,0,0)	3.46(2.18,5.13)	3.55	(2,0,2)	3.82(2.74,4.91)	3.80	(2,0,2)	1.68(1.12,2.39)	1.40	(1,0,2)	2.35(1.59,3.44)	2.07
2005–2006	(1,0,2)	1.42(0.86,2.09)	1.13	(1,0,0)	2.52(1.48,3.67)	2.03	(1,0,0)	3.49(2.34,5.02)	3.51	(2,0,2)	2.10(1.61,2.76)	1.82	(2,0,2)	2.57(1.94,3.51)	2.13
2006–2007	(1,0,2)	1.65(1.19,2.31)	1.36	(1,0,0)	2.53(1.50,3.7)	2.22	(1,0,0)	3.56(2.64,5.06)	2.64	(2,0,2)	2.34(1.87,3.08)	1.88	(2,0,2)	2.86(2.27,3.82)	2.40
2007–2008	(1,0,2)	1.22(0.74,1.85)	1.55	(1,0,0)	1.91(0.91,3.07)	2.07	(1,0,0)	2.56(1.44,4.03)	3.08	**(2,0,2)**	**2.18(1.72,2.89)**	**1.71**	(2,0,2)	2.12(1.48,2.80)	2.08
2008–2009	(1,0,2)	1.07(0.58,1.72)	0.63	(2,0,1)	1.97(1.16,3.01)	1.66	(1,0,0)	2.34(1.49,3.64)	1.66	(2,0,2)	1.58(1.13,2.47)	1.18	(2,0,2)	1.22(0.56,1.96)	0.99
2009–2010	(1,0,2)	1.14(0.49,2.00)	0.78	(1,1,0)	2.31(1.14,3.63)	1.69	(1,0,3)	2.39(1.45,3.78)	1.98	(2,1,3)	2.22(1.63,3.23)	1.68	(2,0,2)	1.99(1.31,2.88)	1.33
2010–2011	(1,0,2)	1.16(0.65,1.94)	0.96	(2,0,2)	4.22(3.31,5.49)	3.34	(1,0,3)	2.73(1.83,3.95)	2.20	(2,0,2)	3.53(2.92,4.44)	3.25	(2,0,2)	1.86(1.20,2.62)	1.53
2011–2012	(1,0,3)	1.04(0.54,1.86)	0.91	(2,0,2)	1.23(0.31,2.5)	1.31	(1,0,3)	2.25(1.41,3.54)	1.70	**(2,0,2)**	**2.39(1.77,3.28)**	**1.64**	(2,0,2)	1.68(1.02,2.66)	1.25
2012–2013	**(2,0,2)**	**4.15(3.66,4.99)**	**3.22**	**(2,0,2)**	**5.5(4.61,6.77)**	**4.52**	**(2,0,1)**	**7.26(6.45,8.58)**	**4.82**	**(2,0,2)**	**6.60(6.05,7.54)**	**4.45**	**(2,0,2)**	**6.28(5.66,7.12)**	**4.48**

**Table 4:** Week 1 ILI rate prediction for HHS regions 6–10 from influenza seasons 2004–2005 to 2012–2013.

	HHS6			HHS7			HHS8			HHS9			HHS10		
2004–2005	(3,0,2)	2.69(1.05,4.09)	2.35	(1,0,2)	1.79(0.44,4.07)	1.43	(2,0,0)	1.65(1.00,3.43)	1.69	**(1,0,2)**	**4.67(3.43,6.09)**	**3.13**	(1,0,0)	1.79(0.20,3.82)	1.84
2005–2006	(2,0,2)	6.39(5.04,7.80)	5.07	(1,1,2)	1.09(-0.36,2.57)	0.77	**(2,0,0)**	**2.65(1.99,3.76)**	**1.87**	**(1,0,0)**	**5.25(3.84,6.97)**	**3.37**	(1,0,0)	2.36(1.08,4.39)	2.41
2006–2007	**(2,0,2)**	**5.48(4.26,7.38)**	**3.86**	(1,1,2)	2.91(1.54,3.77)	2.12	**(1,0,0)**	**1.40(0.64,2.45)**	**0.63**	(1,0,2)	2.66(1.39,4.05)	2.81	(1,0,0)	2.12(0.86,3.86)	1.36
2007–2008	(2,0,2)	4.07(2.53,5.56)	3.94	(1,1,2)	1.77(0.41,2.53)	1.38	(1,0,0)	1.43(0.65,2.37)	0.99	(1,0,2)	3.65(2.57,5.05)	3.65	(1,0,0)	2.54(1.34,4.5)	1.44
2008–2009	**(2,0,2)**	**4.55(3.36,6.00)**	**2.51**	(4,0,0)	0.25(-0.60,1.25)	0.41	(2,0,1)	0.55(-0.18,1.52)	0.34	(1,0,2)	3.14(1.86,4.56)	2.18	(1,0,0)	2.03(0.71,3.83)	1.35
2009–2010	(2,1,0)	3.52(1.80,5.34)	2.69	(2,0,2)	2.07(1.05,3.23)	1.80	(1,0,2)	1.21(0.41,2.08)	0.94	(1,0,0)	4.2(2.93,5.75)	3.09	(0,1,0)	1.99(0.08,3.92)	1.13
2010–2011	(2,0,2)	3.83(2.50,5.70)	3.33	(2,0,2)	2.23(1.28,3.24)	1.68	(1,0,2)	1.02(0.33,1.87)	0.72	(1,0,0)	3.98(2.90,5.53)	3.23	(1,0,0)	2.27(0.87,4.11)	1.44
2011–2012	(2,0,2)	2.36(1.08,3.98)	2.14	(2,0,2)	1.76(0.79,3.16)	1.48	(1,0,2)	1.10(0.43,1.94)	1.05	(1,0,0)	3.55(2.46,4.91)	3.00	(1,0,0)	1.28(0.17,2.95)	1.09
2012–2013	**(2,0,2)**	**9.55(8.25,11.02)**	**7.67**	**(4,0,0)**	**7.01(6.15,8.26)**	**5.81**	(2,0,2)	4.14(3.51,4.93)	3.74	**(1,0,0)**	**4.59(3.55,5.95)**	**3.44**	(1,0,0)	3.19(2.13,4.85)	2.78

Note: 1) Auto-regressive integrated moving average (ARIMA [*p*, *d*, *q*]) method, in which *p* represents the number of auto-regressive terms, *d* is the number of non-seasonal differences and *q* is the number of lagged forecast errors in the prediction equation; 2) 95% prediction intervals were calculated by bootstrapping the model error 5,000 times; and 3) the point estimate and prediction interval were bolded if the observed ILIs rate were not covered by the 95% prediction interval.

### Predicted ILI Rates Lower than Observed

Of 1,530 model predictions from influenza seasons 2004–2005 to 2012–2013 for each HHS region between weeks 44 and week 8, 927 (61%) predicted weekly ILI rates were lower than observed (S2 Table). The percentage of predicted ILI rates that were lower than observed ranged from 44% in 2009–2010 to 67% in 2004–2005. The percentage of predicted ILI rates that were lower than observed was similar across regions (from 58% in Region 10 to 63% in Region 5).

There were 64 of 1,530 predicted ILI rates that were statistically significantly lower than observed (i.e., the observed weekly ILI rate was higher than the upper bound of 95% bootstrapped prediction interval). These 64 predicted values were almost evenly distributed by HHS region. However, seasons 2007–2008 and 2012–2013 contributed 18 and 17 predictions, respectively. Of the 64 predicted values that were significantly lower than observed, the most commonly identified week numbers were weeks 52, 51, 5, 7, and 4, which contributed 12, 9, 8, 7, and 7 values, respectively.

**Fig 1:**
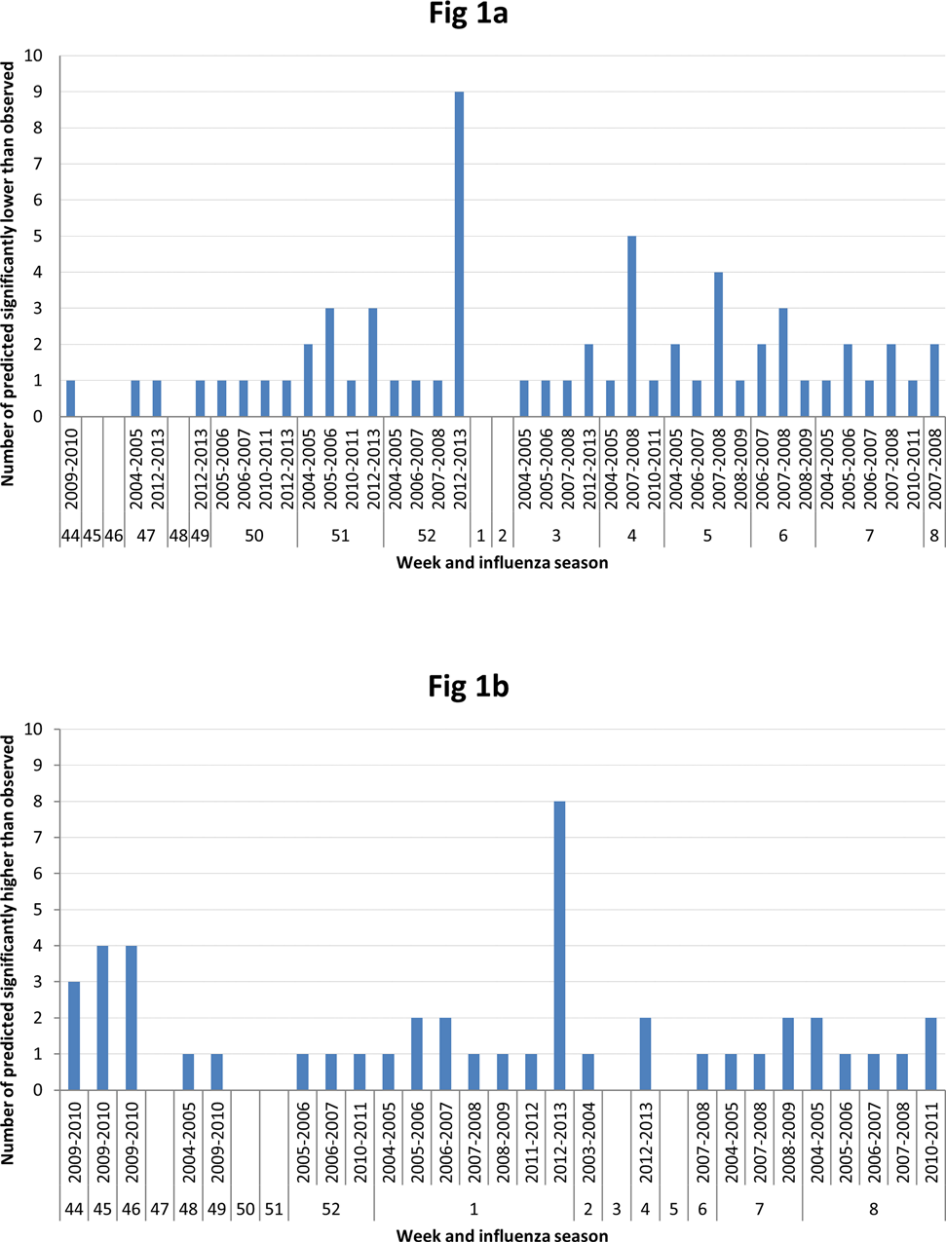
a) Number of predicted that are significantly lower(the upper bound of 95% prediction interval lower than the observed) than observed across influenza season and weeks, b) Number of predicted that are significantly higher(the lower bound of 95% prediction interval higher than the observed) than observed across influenza season and weeks.

Fig 1A illustrates the number of predicted rates that were significantly lower than observed by influenza season and weeks. Weeks 51–52 form an apparent cluster, with a combined 21 (33% of 64) predicted values that is significantly lower than observed. Twelve of the 21 values in this cluster were from influenza season 2012-2013and nine from week 52 (Tables 1 and 2).

### Predicted ILI Rates Higher than Observed

Of 1,530 predicted ILI rates, 38% (579) were higher than the corresponding observed values (S2 Table). Influenza season 2009–2010 had the most predicted ILI rates that were higher than observed (93/1,530; 6%) and influenza season 2010–2011 was the least represented (48/1,530; 3%). There was regional variation in the number of ILI predictions that were higher than observed (36% in region 5 and region 9 to 42% in region 10).

There were 46/1,530 (3%) predictions that were statistically significantly higher than observed (i.e., the observed weekly ILI rate was lower than the lower bound of 95% bootstrapped prediction interval). Influenza season 2009–2010 comprised most of these predictions (12/46; 26%), followed by 2012–2013 (10/46, 22%) and 2004–2005 (5/46, 11%).

Fig 1B displays the 46 predicted ILI rates that are significantly higher than observed by influenza season and week. Most of these predicted values were for week 1 (16/46, 35%). A cluster that accounted for 11 predicted values was seen from weeks 44–46. Influenza season 2012–2013 dominated the contribution from week 1 (Tables 3 and 4), and influenza season 2009–2010 contributed the cluster of predictions from weeks 44–46.

### Model Predictions at Peak and after Peak

While timing of peak-observed ILI activity often differed by HHS region, ILI activity peaked mostly during a single week during some influenza seasons. For example, in influenza season 2009–2010, the peak observed weekly ILI rate occurred in week 44 in all 10 HHS regions. In influenza season 2012–2013, the peak occurred in week 52 in eight of the 10 HHS regions. In seven of the 10 HHS regions, the peak ILI rate occurred in week 7 during influenza seasons 2004–2005 and 2007–2008.

Of 90 (10 HHS regions x 9 seasons) predictions during the peak week ILI activity, 78 predicted ILI rates were lower than observed. In seasons in which the peak week ILI activity occurred in week 7 or earlier, 62 out of 73 predictions for the post-peak week (i.e., the week after the peak ILI week) were higher than observed. There were 53 out of 73 models that had lower peak and higher post-peak predicted ILI rates than were actually observed.

## Discussion

From weeks 44 to week 8 of the influenza seasons investigated, week 52 (during the typical winter holiday break) had the most predicted ILI rates that were significantly lower than observed and week 1 (after the typical winter holiday break) had the most predicted ILI rates that were significantly higher than observed. However, these findings were largely driven by a single influenza season (2012–2013). During 2009–2010, an unusual season due to the emergence of influenza A(H1)pdm09, weeks 44–46 demonstrated a similar phenomenon, with an unusually high number of predicted ILI rates significantly higher than observed.

Influenza seasons 2012–2013 and 2009–2010 share some common characteristics in terms of reported weekly ILI rates by ILINet. First, both seasons had earlier starts than the other influenza seasons we investigated in this study. Secondly, both seasons had a very narrow peak across all U.S. HHS regions; this occurred in weeks 41–42 for influenza season 2009–2010 and week 52 for influenza season 2012–2013. This could be one explanation for our findings. For example, if we look at influenza season 2012–2013 alone, seven of 10 HHS regions reported peak ILI activity in week 52; 13 out of 20 first-week and last-week predictions have either ARIMA(2,0,2), ARIMA(2,0,1), or ARIMA(1,0,2). When time series is used to forecast the peak values along the timeline, the predicted value will depend on the past week or a rolling average of the past two weeks’ values, in most cases. As a result, the forecasted ILI at week 52 will be smaller than the highest observed ILI. However, when the model is trying to forecast the data point next to the peak, the peak observation exerts influence on this prediction, which is then predicted as a value higher than that observed.

**Fig 2:**
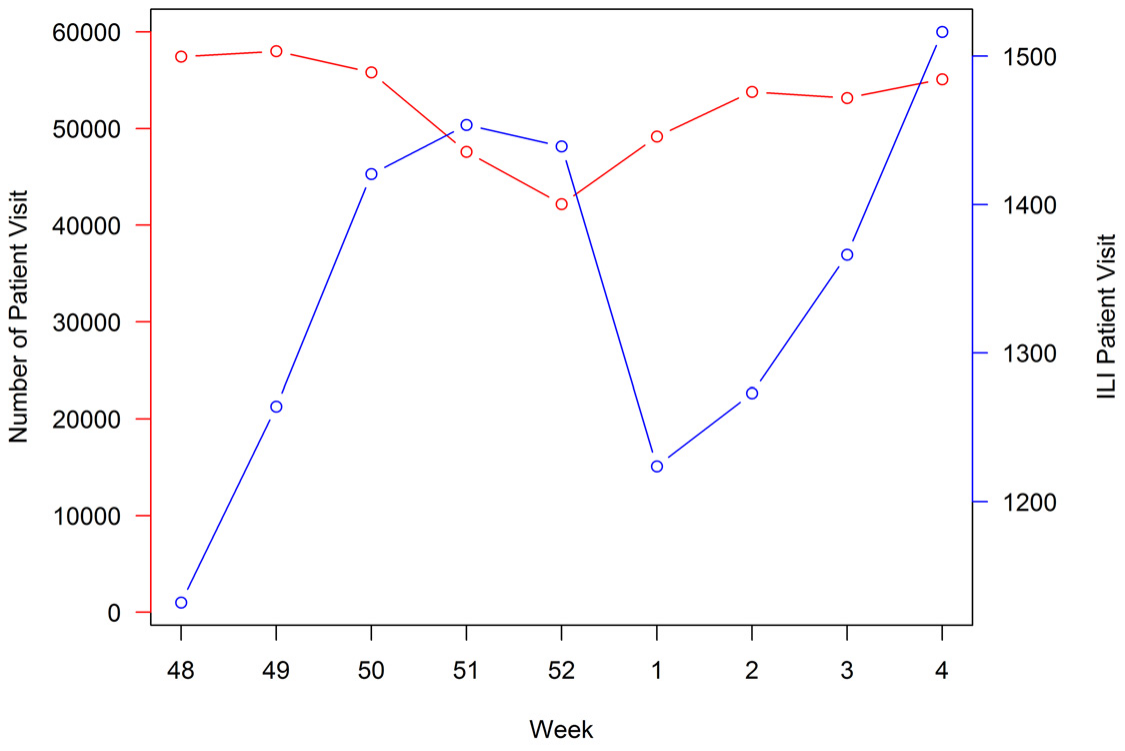
Average total number of patient visits (red line, scales on the left-side y-axis) and average total number of ILI visits (blue line, scales on the right-side y-axis) across all HHS regions between 2003–2004 and 2012–2013 influenza seasons.

Changing daily routines or traveling to other parts of the country during the holiday break could influence the decision to seek care for ILI symptoms. During week 52, the total number of visits reported to ILINet decreases, while the number of ILI-related visits remains the same around the peak, resulting in an elevated proportion of ILI visits (Fig 2). Because ARIMA model predictions for week 52 are based on previous weeks when the total number of visits was higher, the observed ILI rate may be higher than expected. In early January, the observed proportion of ILI may decrease because of the increase in total patient visits. The model prediction in the week after the holiday break is based on previous ILI rates, including the holiday week with the lower denominator of visits; this can lead to predicted ILI rates that are higher than observed. In addition to the changes in the denominator of patient visits during the holiday break, ILI cases requiring medical attention may be more severe than ILI cases reported during non-holiday weeks because of differences in patient care-seeking behavior.

The variability we detected between predicted and observed ILI rates during the winter holiday break of certain influenza seasons may reflect several factors, including a change in social mixing patterns, healthcare-seeking behavior, specific characteristics of the circulating influenza strain, or artifacts of ILINet surveillance data. ILINet surveillance does not capture the full age-specific information; therefore, we could not explore whether our findings are related to age-specific ILI reports or other factors.

We were unable to assess the role of different influenza strains on the predicted ILI rates, although such differences can impact the epidemiology of influenza [12, 13]. Previous infection from one strain can provide full or partial immunity to the same strain, or similar influenza strains, during subsequent exposures. Additionally, certain strains are associated with more or less transmissibility and pathogenicity, affecting illness severity and use of healthcare services. Any of these virus-related factors, as well as co-circulation or serial circulation of different strains, could have influenced our findings.

Our time-series analysis was an ecological approach to describing the relationship between ILINet data and the winter holiday break. As a result, we cannot establish a causal relationship between winter holiday breaks and changes in ILI rates. ILINet provides weekly data on all people with ILI, and since these data do not rely on laboratory confirmation, reported ILI rates likely included people with influenza as well as other acute respiratory diseases. Repeating this analysis using confirmed influenza cases could be informative. Additionally, we divided the winter holiday break into two time frames in this analysis: the last week of the year and the first week of each year. This categorization was made based on our estimation of the typical duration of the winter holiday break, given limited information in the literature as well as the availability of surveillance data by week. Future analyses interpolating daily changes in ILI rates may be warranted. We must also note that our time-series analysis used only 51 and 52 data points for predicting ILI rates for the last week and the first week of the 2003–2004 influenza season, respectively. This could have limited the reliability of our predictions; more data points would have been beneficial.

## Conclusion

In our analysis, we detected high variability in the temporal relationship between winter holiday break and weekly ILI rates across influenza seasons. While overall observed ILI rates during the last week in December were higher than predicted, and observed ILI rates during the first week of January were lower than predicted, these findings were mainly attributable to a specific influenza season 2012–2013. We demonstrated the use of the ARIMA method in conducting this time-series analysis. Additional analyses using this method and others to incorporate environmental factors or virological data may better clarify the relationship between influenza activity and the winter holiday break.

## Supporting Information

S1 Fig Model selection and assumption check. The ARIMA model selection and assumption check for the last-week prediction of 2004 in HHS region 4: a) autocorrelation function indicated a wide range of lagged error terms (*q* term in ARIMA [*p*,*d*,*q*]); b) partial autocorrelation function indicated the number of auto-regressive terms (*p* term in ARIMA [*p*,*d*,*q*]) was either 1 or 2; c-d) based on Bayesian information criterion, ARIMA(*2*,*0*,*2*) was selected for the time-series fitting and histogram and quantile-quantile plot assessed the normality assumption for time-series residuals.
(TIF)

S2 Fig Last-week ILI rate prediction for HHS region 4. Last-week ILI rate prediction for HHS region 4 was based on previously observed weekly ILI rates and previously fitted last-week ILI rate. A solid blue line represented weekly ILI rates reported by CDC ILInet, a solid red line represented fitted weekly ILI rates. Blue dots and red dots represented the observed and predicted week-52 ILI rates, respectively, with ARIMA models for the following years: a) 2004 last-week prediction by ARIMA(*2*,*0*,*2*); b) 2006 last-week prediction by ARIMA(*2*,*0*,*2*); and c) 2008 last-week prediction by ARIMA(*2*,*0*,*2*).
(TIF)

S1 Model Fitting (DOCX)

S1 Table Bayesian information for the candidate ARIMA models to forecast the 2004 week-52 ILI rate. (DOCX)

S2 Table ARIMA models and predictions for week 44 to week 8 from influenza season 2004–2005 to 2012–2013. (XLSX)
