# Supplementary figures and images for: Comparing Observed with Predicted Weekly Influenza-Like Illness Rates during the Winter Holiday Break, United States, 2004-2013
Source: PLoS One. 2015 Dec 9;10(12):e0143791. doi: 10.1371/journal.pone.0143791 (PMC4674102; doi:10.1371/journal.pone.0143791)

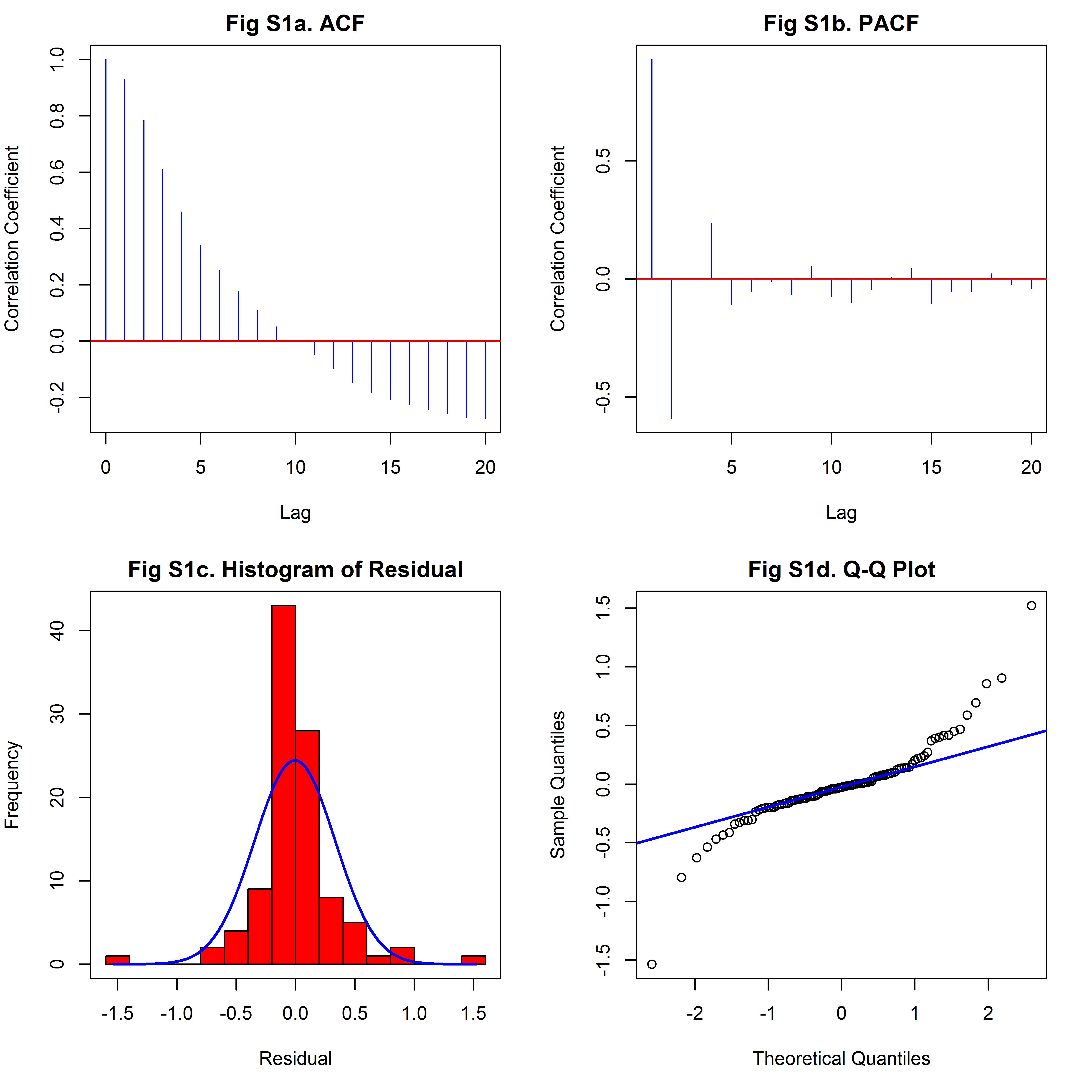

Supplement: S1 Fig — The ARIMA model selection and assumption check for the last-week prediction of 2004 in HHS region 4: a) autocorrelation function indicated a wide range of lagged error terms (q term in ARIMA [p,d,q]); b) partial autocorrelation function indicated the number of auto-regressive terms (p term in ARIMA [p,d,q]) was either 1 or 2; c-d) based on Bayesian information criterion, ARIMA(2,0,2) was selected for the time-series fitting and histogram and quantile-quantile plot assessed the normality assumption for time-series residuals. (TIF) [file pone.0143791.s001.tif]

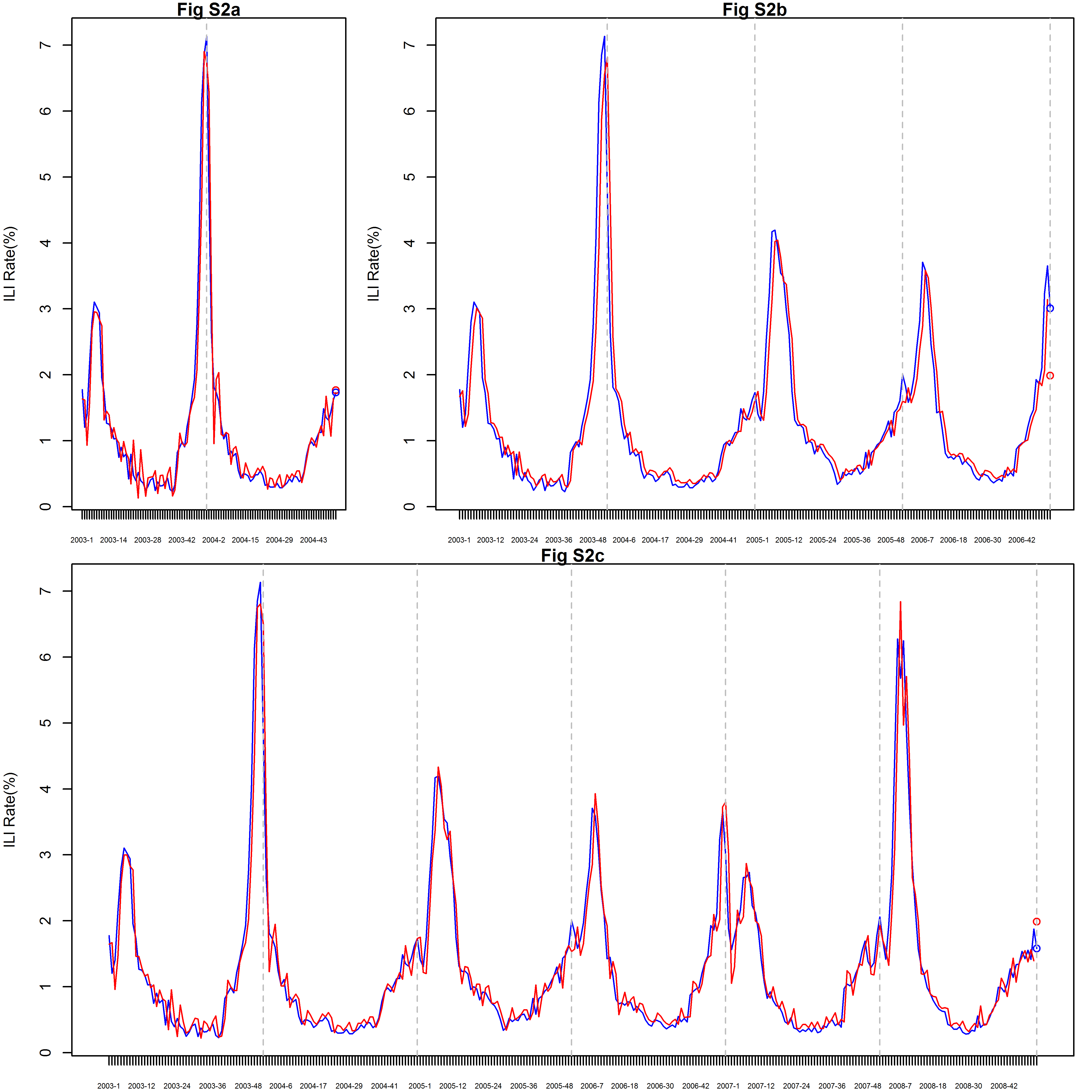

Supplement: S2 Fig — Last-week ILI rate prediction for HHS region 4 was based on previously observed weekly ILI rates and previously fitted last-week ILI rate. A solid blue line represented weekly ILI rates reported by CDC ILInet, a solid red line represented fitted weekly ILI rates. Blue dots and red dots represented the observed and predicted week-52 ILI rates, respectively, with ARIMA models for the following years: a) 2004 last-week prediction by ARIMA(2,0,2); b) 2006 last-week prediction by ARIMA(2,0,2); and c) 2008 last-week prediction by ARIMA(2,0,2). (TIF) [file pone.0143791.s002.tif]
