# Supplementary material for: Comparing Observed with Predicted Weekly Influenza-Like Illness Rates during the Winter Holiday Break, United States, 2004-2013
Source: PLoS One. 2015 Dec 9;10(12):e0143791. doi: 10.1371/journal.pone.0143791 (PMC4674102; doi:10.1371/journal.pone.0143791)
Supplement: S1 Model Fitting — (DOCX) [file pone.0143791.s003.docx]

**S1. Model Fitting.**

We used the 2004 week-52 prediction in HHS region 4 as an example to demonstrate the procedures implemented and results of our model selection. The autocorrelation function (ACF) for the weekly ILI rate (Fig S1a, ACF) suggested that the number of lagged error terms can have a wide range, while the partial auto correlation function (Fig S1b, PACF) indicated that the number of auto-regressive terms is either 1 or 2. Among many candidate models (S1 Table), ARIMA (2, 0,2) had the minimum value of Bayesian information and, therefore, was selected in the forecasting step. The Ljung-Box test resulted in a p- value of 0.95, up to 6 lags, which supported the null hypothesis of zero correlations among the residuals. Furthermore, the distribution of model residuals demonstrated certain normality (Figs S1c and d).

The forecasting step using ARIMA (2,0,2) resulted in a predicted ILI rate of 1.78% (95% bootstrapped prediction interval 1.20%-2.65%) for the last week in 2004 for HHS region 4. In contrast, the observed ILI rate for the same week, year, and region was 1.73%. Figure S2 illustrates examples of the observed and predicted curves in HHS region 4 for 2004 (Fig S2a), 2006 (Fig S2b), and 2008 (Fig S2c), in which blue dots represent the observed last-week ILI rate reported by CDC ILINet and red dots represent the forecasted last week ILI rate by the ARIMA model.
