# Supplementary material for: Comparing Observed with Predicted Weekly Influenza-Like Illness Rates during the Winter Holiday Break, United States, 2004-2013
Source: PLoS One. 2015 Dec 9;10(12):e0143791. doi: 10.1371/journal.pone.0143791 (PMC4674102; doi:10.1371/journal.pone.0143791)
Supplement: S1 Table — (DOCX) [file pone.0143791.s004.docx]

**S1 Table. Bayesian information for the candidate ARIMA models to forecast the 2004 week-52 ILI rate.**

| ARIMA(*p,d,q*)* | Bayesian Information | ARIMA(*p,d,q*)* | Bayesian Information |
| --- | --- | --- | --- |
| (0,0,0) | 355.58 | (2,0,2) | **92.30** |
| (0,0,1) | 250.43 | (1,0,1) | 122.76 |
| (2,0,3) | 101.92 | (1,0,2) | 97.49 |
| (1,0,0) | 151.17 |  |  |

**p* represents the number of previous observations upon which the current observation linearly depends, *d* is the order of differencing, and *q* is the number of proceeding estimation errors taken into account when estimating the next time-series value.
